# Impaired Gastric Myoelectrical Reactivity in Children and Adolescents with Obesity Compared to Normal-Weight Controls

**DOI:** 10.3390/nu10060699

**Published:** 2018-05-31

**Authors:** Katja Weimer, Helene Sauer, Bjoern Horing, Francesco Valitutti, Nazar Mazurak, Stephan Zipfel, Andreas Stengel, Paul Enck, Isabelle Mack

**Affiliations:** 1 Clinic for Psychosomatic Medicine and Psychotherapy, University Hospital Ulm, 89081 Ulm, Germany; katja.weimer@uni-ulm.de; 2 Department of Psychosomatic Medicine and Psychotherapy, University Medical Hospital Tübingen, 72076 Tübingen, Germany; helene.sauer@medizin.uni-tuebingen.de (H.S.); nazar.mazurak@med.uni-tuebingen.de (N.M.); stephan.zipfel@med.uni-tuebingen.de (S.Z.); andreas.stengel@med.uni-tuebingen.de (A.S.); paul.enck@uni-tuebingen.de (P.E.); 3 Institute of Systems Neuroscience, University Medical Center Hamburg-Eppendorf, 20246 Hamburg, Germany; b.horing@uke.de; 4 Pediatric Gastroenterology and Liver Unit, Department of Pediatrics, Sapienza University of Rome, 00161 Rome, Italy; francesco.valitutti@uniroma1.it; 5 Department for Psychosomatic Medicine, Charité Center for Internal Medicine and Dermatology, Charité-Universitätsmedizin, Corporate Member of Freie Universität Berlin, Humboldt-Universität zu Berlin, and Berlin Institute of Health, Berlin, 12200 Berlin, Germany

**Keywords:** obesity, stress, gastric myoelectrical reactivity, brain-gut axis, autonomic nervous system, psychosomatic

## Abstract

Obesity often has its onset in childhood and can be accompanied by various comorbidities such as functional gastrointestinal disorders and altered gastric myoelectrical activity (GMA). This study investigates whether obesity in childhood and adolescence is already associated with altered GMA, and whether an inpatient weight loss program affects GMA. Sixty children with obesity (OBE) and 27 normal-weight children (NW) (12.9 ± 1.7 years; 51% female) were compared for their GMA at rest, after a stress test, and after a drink-to-full water load test. A continuous electrogastrogram (EGG) was recorded and analyzed with respect to gastric slow waves and tachygastric activity. OBE were examined upon admission (T1) and before discharge (T2) following an inpatient weight loss program; NW served as control group. Compared to NW, children with obesity showed flattened GMA as indicated by lower tachygastric reactivity after stress and water load test at T1. Data of OBE did not differ between T1 and T2. EGG parameters were associated neither with sex, age, and BMI nor with subjective stress and food intake. Children with obesity show impaired gastric myoelectrical reactivity in response to a stress and water load test compared to normal-weight controls, which does not change during an inpatient weight loss program.

## 1. Introduction

Obesity is an increasing problem worldwide and often has its onset in childhood. The World Health Organization (WHO) reports that 6% of girls and 8% of boys between 5 and 19 years worldwide were obese in 2016 [1]. Children with obesity are at high risk of becoming obese adults and developing a variety of physical and mental comorbidities such as cardiovascular diseases, musculoskeletal disorders, or diabetes [1]. Furthermore, there is a high overlap of obesity with functional gastrointestinal disorders (FGIDs) [2] as well as dysfunctions of the autonomic nervous system (ANS) [3,4].

Related to a higher prevalence of FGIDs in children with obesity or overweight compared to normal-weight children [2], it has been questioned if obesity is the cause or consequence of FGIDs [5]. Ho and Spiegel (2008) discuss several pathways by which FGIDs could enhance the risk for obesity such as side effects from taking proton pump inhibitors (PPI), binge eating disorders, variations in gastric volume and emptying rate, or alterations in GI neuropeptide functions [5]. However, other authors discuss that the link between obesity and FGIDs could be a dysfunction of the ANS [4,6]. The latter is assessed mostly through recordings of electrocardiograms (ECG) during rest and stress tests, and parameters of heart rate variability (HRV) are analyzed. Differences in HRV between obese or overweight adults and children compared to normal-weight controls have been reported repeatedly: most studies found an autonomic imbalance towards an increased sympathetic and a decreased parasympathetic activity or reactivity in obese subjects [3,4,7,8,9,10,11]; however, results are inconclusive [4]. The ANS is involved in all organ functions and, therefore, such general changes in activity and reactivity of the two branches of the ANS can lead to impaired functions in multiple ways. Effects are best studied for HRV for which an autonomic imbalance has been indicative for a higher risk of severe cardiovascular disorders [12].

Similar to HRV, gastric myoelectrical activity (GMA) and reactivity (GMR) are affected by the ANS [13,14]. Thus, it is not surprising that obesity in adulthood is also associated with altered GMA, so called dysrhythmic slow waves [15], that are also related to other disorders affecting the stomach, e.g., motility disorders or gastroparesis in diabetes [16]. There is only one study investigating GMA in obese children that reported no differences between children with obesity and controls with respect to their GMA at rest and after two different types of meals [17]. However, it is unknown whether obesity in childhood is accompanied by dysrhythmic slow waves in response to challenging situations such as stress-provoking tasks or acute gastric dilation in a drink-to-full water load task.

Gastric slow waves are electrical potentials propagating along the stomach, generated by pacemaker cells (interstitial cells of Cajal, ICC) and smooth muscle cells, and can be coupled with gastric motility and contractions. In healthy humans, GMA is influenced mainly by two factors: (1) the stomach’s content such as the composition of meals and the ingested volume, and (2) the ANS [15]. Most results derive from experimental studies in adults, but the relationship between these factors in children and adolescents with obesity is largely unknown. As GMA is associated with the ANS along with the ingested volume and composition of food, we compare the GMA and GMR of children with obesity to lean children during a resting period, after a stress test, and after a drink-to-full water load test.

We hypothesized that (1) obese children do not differ from normal-weight children during a resting period (according to the literature), but (2) that an imbalance in the ANS could result in differences in response to a stress task and a water load test. Additionally, we hypothesized that (3) an inpatient rehabilitation program leads to a normalization of GMA at rest and GMR in response to a stress task and a water load test in obese children towards values of normal-weight children.

## 2. Materials and Methods 

### 2.1. Study Population and Design

The study protocol was approved by the Ethics Committee of the University Hospital Tübingen, Germany. This study was registered at the German Clinical Trials Register (DRKS) with the clinical trial number DRKS00005122. The analysis presented here was conducted as part of the DROMLIN study (preDictor Research in Obesity during Medical care—weight Loss in children and adolescents during an INpatient rehabilitation) [18], for which the overall sample size was calculated. The aim of the DROMLIN study was to identify predictors which play a role in successful weight loss and body weight maintenance in children and adolescents. For the comparison of electrogastrogram (EGG) data between children with obesity (OBE) at admission (T1) and children with normal-weight (NW), a sample size of *n* = 48 allows us to test for medium to high effect sizes of d = 0.8 (Mann–Whitney U test, α = 0.05, power = 0.80, allocation ratio 1:2) as calculated with G*Power Version 3.1.9.2 [19], which was achieved in all comparisons.

Children and parents were informed about the study purpose during the admission interview and asked to provide written consent prior to inclusion. In short, OBE with a body mass index (BMI) above the 90th percentile of the age and sex specific norms [20], aged between 9 and 17 years and an indication for inpatient weight loss intervention at the Children Rehabilitation Hospital for Respiratory Diseases, Allergies and Psychosomatics, Wangen i.A., Germany were included. Children between the 90th and 97th percentile are classified as overweight, above the 97th percentile as obese [20]. Children in this program are treated by a multidisciplinary team in accordance with the latest developments in medicine, and the program operates in close cooperation with regional educational institutions, such as the Obesity Academy in Baden-Württemberg, Germany (Adipositas-Akademie Baden-Württemberg e.V.). Children are treated in small therapeutic groups with peers of the same age and housed in residential units situated on a park-like hospital ground. The clinic has its own school with regular classes for all types of curricula. The obesity program focuses on exercise and a balanced diet in order to achieve weight loss. No medication is applied for weight loss. Smoking is forbidden during the inpatient stay. The obesity treatment concept at the rehabilitation hospital is described in detail elsewhere [18]. Additionally, sex- and age-matched NW with a BMI between the 10th to 90th BMI percentile [20] from the catchment area of the University Hospital Tuebingen, Germany served as control group which was tested only once. Exclusion criteria were severe psychological comorbidities, linguistic or intellectual limitations, diabetes, malignant tumors, systemic disorders, or severe cardiovascular diseases. 

Medication, comorbidities, and gastrointestinal (GI) symptoms were assessed using questionnaires for children and their parents. A sum score for GI symptoms was calculated from six items of the Children’s Somatization Inventory [21] asking for symptom severity of nausea or stomach upset, obstipation, diarrhea, abdominal pain, vomiting, and flatulence during the last two weeks. Answers are rated on a scale from 0 = “not at all” to 4 = “very” resulting in total sum scores between 0 and 24.

The full experimental setup was already described in previously published articles [4,18]. Here, we describe the relevant setup for the EGG experiments only. Testing took place in the morning (10:00–11:30 a.m.) about 2 h after breakfast. The inpatient group was tested twice: upon admission (T1) and prior to discharge (T2). Duration of hospital stay was 38 ± 10 days (Mean ± SD) (minimum-maximum: 16–70 days). At testing days, each child completed a standardized EGG recording session with baseline (20 min), mental stress (5 min), post-stress recovery phase (20 min), drink-to-full water load test (5 min), and a post-water load recovery phase (20 min). During baseline and recovery recordings, children were asked to sit comfortably in a chair, avoid movements or speaking and were allowed to watch a quiet movie of their choice depending on their age and interests. During the mental stress task, children were asked to count backwards in steps of 7 from 1000 (age 13–17), 300 (age 11–12), or 100 (age 9–10 or children with deficiencies in math) in front of a camera with a red blinking light (comparable to tasks of the Trier social stress test, [22]). They were instructed to count as quickly as possible and told that mistakes were recorded. Additionally, they were told that the test was a competition between their peers and the winner only would receive a gift coupon. After the stress task, children were asked to rate their subjective stress experience using a 6-point scale where 1 corresponded to “absolutely not stressed” and 6 to “absolutely stressed” (according to German school grades). Participants were then asked to sit for another 20 min for the post-stress session, during which they were allowed to continue to watch the movie. Afterwards, children were instructed to drink as much water as possible within 3 min, or until feeling too full to continue (see below). Finally, they were asked to sit for another 20 min for the post-water load recording phase, during which they were allowed to continue to watch the movie [4].

### 2.2. Dietary Intake and Water Load Test

Prior to the EGG measurements the dietary intake of the last meal (breakfast) was assessed by a semi-structured interview. The interview was conducted by a trained nutrition scientist. The participants were fasted for two hours before the water load test was conducted as reported previously [23]. Briefly, the participants were asked to drink as much water as possible within 3 min, or until feeling too full to continue. The water was portioned out in 250 mL servings from a 1.0 liter jug of tap water at room temperature. The volume of water remaining was determined using a measuring cup with a 10-mL level of accuracy.

### 2.3. Electrogastrography

Gastric myoelectrical activity was recorded by an EGG for which three skin electrodes were placed above the stomach [24], and connected to a Biolog device with Fetrodes technology (UFI, Morrow Bay, CA, USA). The EGG was recorded with a sampling rate of 10 Hz (filter settings: band-pass filter with a low cutoff of 0.0175 Hz and a high cutoff of 0.33 Hz both with 12 dB per oct roll-off), and stored for offline analysis. Recordings were screened visually for artifacts. Criteria for artifacts were signals with improbable amplitudes (±1000 μV) for myoelectrical activity of the stomach, and fast and sudden onset that did not fit to the surrounding signals. Three segments of at least 4.27 min length from recordings at baseline, after the stress test, and after the drink-to-full water load test were selected for analysis. Selected EGG data were analyzed with a fast Fourier transformation procedure (FFT; custom software using Prime Factor FFT for Windows, version 3.03, Alligator Technologies, Costa Mesa, CA, USA) and a spectral resolution of 0.29 cycles per minute (cpm). A frequency range between 2.03 to 4.06 cpm was regarded as normal gastric activity (normogastria) and a range between 4.35 to 8.99 cpm as tachygastria. Spectral resolution and frequency bands depend on the sampling rate which was higher than in most other studies (mostly 5 Hz), and therefore deviate from standard descriptions [25]. The percentage spectral power was obtained from the total range of 0.87 to 14.79 and the ratio between the percentage of the normogastria and the tachygastria band (EGG ratio) as an indicator for the balance of these bands was calculated. Dysrhythmic slow waves, i.e., the interruption of the normal 3-cpm activity of the stomach and a shift towards tachygastria indicated by a decrease of the EGG ratio has been repeatedly associated with impaired gastric activities in obesity [15].

### 2.4. Statistics

Data were analyzed using IBM SPSS Statistics for Windows, Version 24.0 (IBM Corp. Released 2016. Armonk, NY, USA: IBM Corp.). Normally distributed data are presented as mean ± standard deviation. Non-normally distributed data are presented as median (interquartile range) and the EGG variables additionally by mean ± standard deviation. Differences between OBE T1 and NW were calculated using unpaired *t*-tests (height, BMI, BMI-SDS), Chi^2^ test (sex) or Mann–Whitney U-tests if data were not normally distributed (age, weight, waist circumference, EGG variables). Differences between OBE T1 and OBE T2 were analyzed with paired *t*-test (BMI, BMI-SDS) or Wilcoxon signed-rank test if data were not normally distributed (weight, waist, EGG variables). We chose a longitudinal hierarchical linear modeling (HLM) approach for analysis of EGG data [26]. HLM is robust to univariate deviations from normality, provided a normal residual distribution. In particular, using HLM allowed us to handle missing data more flexibly, as the number of repeated measures can differ in HLM. HLM provides estimates adjusted for the degree of nesting or correlated errors. Centering for within- and between-persons predictors was performed according to recommendations [27].

For each EGG parameter, two separate multilevel analyses were performed, with additional analyses as post hoc comparisons. Model 1 included period (baseline, stress test, water load test) and group (OBE T1 versus NW) as predictors, as well as their interaction. Post hoc comparisons were performed for both groups separately, using period only. Model 2 included the OBE group only, and used period and day (T1 versus T2) as predictors, as well as their interaction. All HLM analyses were performed as random intercept models only, as inclusion of repeated measures as random effects did not significantly contribute to explained variance. Lower-order predictors (period, day) were entered first, followed by higher-order predictors (group), where applicable. For each EGG parameter (normogastria, tachygastria, EGG ratio) respectively, within-person variance constituted 67.6, 68.1, and 85.4% of the total variance, while between-person variance constituted the remaining 32.4, 31.9, and 14.6%. Wilcoxon signed-rank tests were used as post-hoc tests. We used Spearman correlations to analyze associations between EGG variables, variables of sample description, subjective stress experience and food intake variables, because in most analyzed pairs, at least one variable was not normally distributed.

EGG recordings are very sensitive due to body movements, speech, laughter, or cough. Therefore, children had to sit quietly three times (at baseline, after the stress test, and after the drink-to-full water load test) for 20 min which was difficult for them and finally led to high dropout rates in EGG data during the three recording periods. Children with and without data dropouts did not differ in any other variables such as sex, age, height, weight, BMI, BMI-SDS or waist circumference, food intake, or existing EGG variables (all *p* > 0.05). For EGG data analyses, we therefore used all available data for each analysis leading to deviating sample sizes for each analysis.

In order to control for multiple testing, the *p* values were adjusted to account for false discovery rate (FDR) [28] when multiple comparisons were performed, and only FDR-adjusted *p* values are reported. FDR *p*-value adjustments were performed separately for the different group comparisons (OBE T1 versus OBE T2 separately from OBE T1 versus NW) but across within comparisons ((a) baseline, post-stress, post-water and for each normogastria, tachygastria, EGG ratio which corresponds to 9 *p* values per group; (b) correlations of EGG data with sex, age, body height, body weight, BMI, BMI z-score, weight circumference, and GI symptoms which corresponds to 8 *p* values per group; (c) all values regarding breakfast which corresponds to 9 *p* values per group). FDR-adjustment was not performed for main analyses (HLM), and single variables (e.g., stress ratings).

*p* values and FDR-adjusted *p* values of < 0.05 were considered as statistically significant.

## 3. Results

### 3.1. Study Population

Of the 60 OBE, data of 58 OBE (3 overweight, 55 obese) at T1 and of 53 OBE at T2 were analyzed; dropouts were due to quality of EGG data that was insufficient for any recording period (*n* = 2 excluded at T1 and T2), and due to early treatment termination (additional *n* = 5 at T2). OBE lost on average 4.3 ± 2.5 kg (min-max: (−13.0)–(+1.1) kg) of body weight (*p* < 0.001) and the BMI z-score reduction was −0.2 ± 0.1 units (*p* < 0.001). The BMI z-score is a measure of relative weight adjusted for child age and sex. The average time between investigations was 26.4 ± 8.2 days. The reference group consisted of 26 NW (*n* = 1 dropout due to EGG data quality). The characteristics of the study population are given in Table 1. At T1, OBE were significantly taller and heavier and had higher waist circumferences compared to NW, resulting in significantly higher BMI and BMI z-score values. OBE and NW did not differ in GI symptoms score which was low in both groups.

Neither sex and age, nor body height, weight, BMI, BMI z-scores, waist circumference, and GI symptoms correlated with any of the EGG parameters (all FDR > 0.05).

All OBE were diagnosed with additional comorbidities with the most prevalent being: adjustment disorders (*n* = 15), disorders related to attention, hyperactivity and concentration (*n* = 13), asthma (*n* = 11), hypertonia (*n* = 6), enuresis and/or encopresis (*n* = 6), depressive disorders (*n* = 6), and lipometabolic disorders (*n* = 4). Ten OBE took medication to treat those conditions (*n* = 4: L-thyroxine; *n* = 3: asthma medications; *n* = 2: nasal sprays; *n* = 1 for each Desmotabs^®^ and Medikinet^®^ retard). No such diagnoses were present in NW, but five children took medications due to allergic reactions without asthma (*n* = 3: medication against allergies with no further specification; *n* = 2: nasal sprays).

### 3.2. Comparison between OBE T1 Versus NW

Table 2 shows EGG data for OBE at T1 and NW for the normo- and the tachygastria bands, and the ratio between those two bands. HLM analyses showed no main effects for period or group in any of the EGG parameters, but a significant interaction of period x group for all three parameters (Model 1 in Table 3). This interaction is based on the fact that there is no main effect of period in OBE T1 (n.s. for all EGG parameters), but in NW for all three EGG parameters (normogastria: estimate = −2.37 (SEM = 0.74), *p* = 0.003; tachygastria: 2.14 (0.66), *p* = 0.003; EGG ratio: −0.18 (0.06), *p* = 0.003). Furthermore, within-subject comparisons with Wilcoxon signed-rank tests of EGG parameters between baseline and the stress and the water load tests to assess GMR showed no differences in OBE (all post hoc tests: FDR > 0.05). In NW, Wilcoxon signed-rank tests showed that tachygastric activity significantly increased and the EGG ratio significantly decreased from baseline to after the stress test (Z = −2.54, FDR = 0.034 and Z = −2.21, FDR = 0.048, respectively). Furthermore, compared to baseline, normogastria was significantly lower and tachygastria significantly higher after the water load test (Z = −2.27, FDR = 0.048 and Z = −2.55, FDR = 0.034), and therefore EGG ratio was significantly lower (Z = −2.55, FDR = 0.034), too. There were no significant differences between EGG parameters after the stress test compared to the water load test.

**Figure 1:**
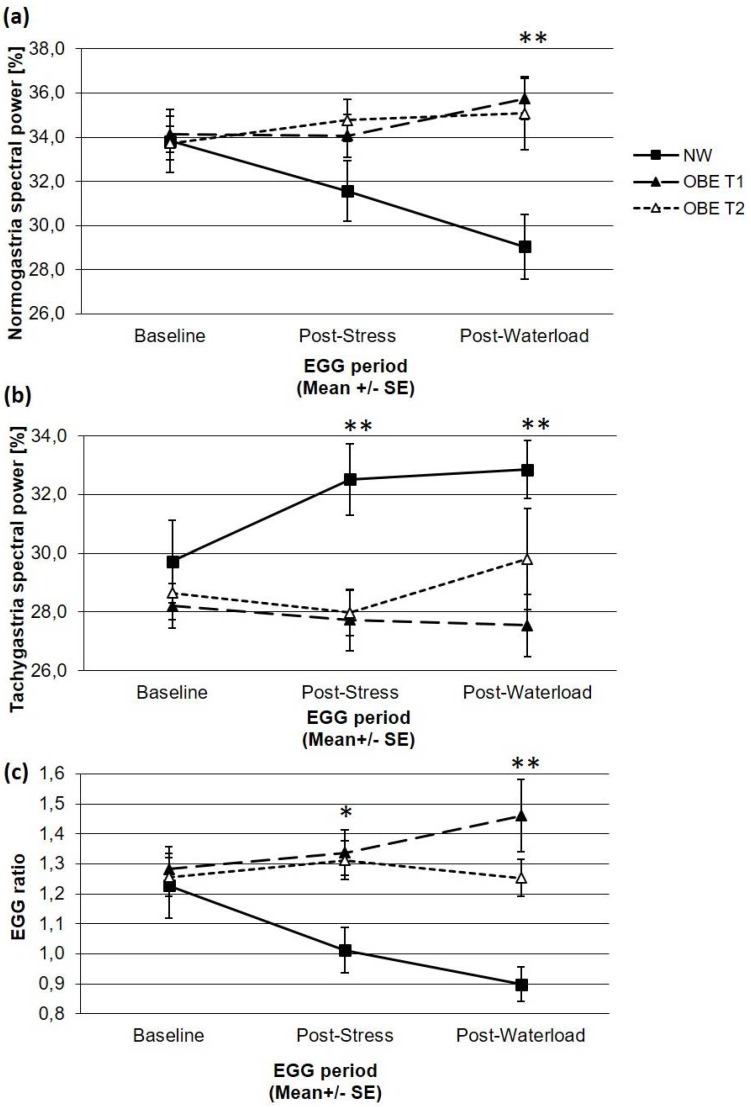
EGG parameters of gastric myoelectrical activity in children with obesity (OBE) before (T1) and after (T2) weight loss and normal-weight control children (NW), depicted as (**a**) normogastria spectral power (%), (**b**) tachygastria spectral power (%), and (**c**) the ratio between normogastria and tachygastria (comparisons between OBE T1 and NW: * FDR < 0.05, ** FDR < 0.01).

OBE and NW did not differ at baseline, but OBE had significant lower tachygastric activity and a higher normo- to tachygastria ratio than NW after the stress test. Furthermore, OBE showed significantly higher normogastric, but lower tachygastric activity than NW after the water load test, resulting in a significantly higher EGG ratio (Figure 1).

### 3.3. Comparison between OBE T1 and OBE T2

HLM analyses showed no main effects or interaction for period or day (T1 versus T2) in OBE in any of the EGG parameters (Model 2 in Table 3). Gastric myoelectrical activities did not differ between T1 and T2 in OBE neither at baseline nor after the stress or the water load test as shown by post-hoc Wilcoxon signed-rank tests. Table 4 shows EGG data for OBE T1 vs. OBE T2 for the normo- and the tachygastria bands, and the EGG ratio between those bands.

### 3.4. Stress, Food Intake, and Water Load Test

The descriptive data and test statistics of possible confounding variables such as stress experienced during the stress task and food intake at breakfast two hours ahead of the baseline measurements are presented in Table 5. At T1, OBE reported significantly higher stress compared to NW as well as compared to T2. None of the food intake parameters of Table 5 correlated with the water load volume. Detailed results of the water load test for OBE T1 versus NW were reported in a previous paper [22]. In short, the intake of water until the onset of satiety was in OBE at T1 450 (345–600) mL, in OBE at T2 410 (310–560) mL and in NW 400 (300–450) mL. Significant group differences for water intake volume only existed between OBE at T1 and NW (U = 521, *p* = 0.024).

There were no correlations between stress rating, food volume and composition with EGG variables (all FDR > 0.05) indicating that neither the stress experienced nor breakfast and the volume of water drank during the water load test affected gastric myoelectrical activity.

## 4. Discussion

We compared children and adolescents with overweight and obesity, who took part in an inpatient weight loss program, with normal-weight children to investigate their gastric myoelectrical activity at rest and in response to a mental stress task and a drink-to-full water load test at admission and discharge. According to the literature and our first hypothesis, GMA activity as measured by EGG parameters did not differ between OBE and their normal-weight age- and sex-matched controls at baseline two hours after a breakfast. As hypothesized, OBE and NW showed different GMR in response to a stressful task and to a water load test. After the stress test, OBE showed significantly lower tachygastria and, related to this, a higher EGG ratio compared to NW. Those differences occurred due to the fact that NW responded to the stress test in an expected manner, namely with a significant increase of tachygastria and a lowered EGG ratio whereas EGG parameters of OBE did not change from baseline to the stress test. Furthermore, responses of NW and OBE continued after the water load test: OBE did not respond with any changes in EGG parameters compared to the baseline or to the stress task, whereas NW showed significantly higher tachygastria and a lower EGG ratio compared to the baseline, but no change compared to the stress task. Overall, the GMR of OBE was flattened during the course of the tests compared to responses of NW. Also, we investigated if the GMA and GMR in obese children will adapt towards values shown by the normal-weight controls, but EGG parameters of OBE did not change from admission to discharge of their inpatient weight loss program.

EGG recordings are based on the fact that the activity of the stomach is substantially influenced by electrophysiological processes. The muscular wall of the stomach contains pacemaker cells (ICC) which produce electrical potentials propagating along the stomach, called gastric slow waves, at a rate of three cycles per minute (3 cpm) all the time. Furthermore, the muscular wall is innervated by parasympathetic fibers as well as sympathetic splanchnic nerves modulating the electrical potentials. After ingestion of food or beverages, the fundus of the stomach relaxes due to vagal efferent influences and release of acetylcholine which (can) lead to a coupling of gastric slow waves and spike potentials to form gastric contractions [25]. Due to the strong influence of the ANS—i.e., parasympathetic and sympathetic activity—on GMA, the latter can respond to stress which is characterized by an increase of sympathetic and decrease of parasympathetic activity, leading to an interruption of gastric slow waves and an increase of tachygastria, which has been shown in experimental studies with healthy participants [13,14]. Therefore, healthy GMA is expected as higher percentage of normogastria (3 cpm) compared to tachygastria at rest, and GMR as an increase of tachygastria in response to stress, and an increase of normogastria in response to a water load test. In our study, healthy NW responded in the expected manner with an increase of tachygastria after the stress test compared to the baseline, but did not change after the water load test. Probably, the effects of the stress test with a withdrawal of parasympathetic activity on the GMA was still present preventing the reappearance of gastric slow waves. In contrast, EGG parameters did not change from baseline to the stress and the water load test in OBE that could be interpreted as low influence of the ANS on GMA and GMR in general. In patients with altered autonomic reactivity, GMA and GMR could be altered, too, e.g., a withdrawal of parasympathetic activity could prevent a coupling of gastric slow waves and spike potentials so that gastric contractions cannot occur [25]. It has been repeatedly shown that patients with obesity have an altered reactivity of the ANS compared to normal-weight controls as assessed by HRV, mostly with a shift toward sympathetic activation and/ or parasympathetic withdrawal [3,4,7,8,9,10,11]. However, the opposite has also been reported and it has been speculated that a lower sympathetic activation at rest could be a risk factor for weight gain due to reduced energy expenditure [29].

Consistent with the latter, an explanation for the failure of the normal response in OBE delivers the so-called “selfish brain theory” [30,31]. This theory assumes that many humans decrease their stress reactivity upon chronic exposure to a stressful environment. This habituation may protect the body from psychological and physiological effects of chronic stress. In this theory, the brain is the key player which has to organize its appropriate supply with energy under conditions of increased demands such as acute psychosocial stress. Therefore, the cerebral insulin suppression mechanism is important for this regulation. The central point of this mechanism is that the brain prevents storage of energy in peripheral tissues and thus, enhances its own glucose supply. In this context, obesity has been discussed as a result of “adaptive phenotypic plasticity” allowing optimized survival in stressful environments [30]. In fact, it was shown that under stress exposure the hypothalamic–pituitary–adrenal axis reactivity was low and no cerebral insulin suppression occurred in obesity [32]. According to the selfish brain theory, compensatory for the failure of cerebral insulin suppression, food intake is increased to maintain the brain energy demands [32,33]. In line with this theory, OBE in comparison to NW failed to react with a physiological EGG response under stress conditions despite higher scorings of perceived stress (see below) in the stress test which may contribute to safe energy in the periphery.

To control for possible confounding variables, we assessed food and beverage intake during breakfast two hours ahead of the baseline measurements, the subjective stress experienced during the stress test, and the water volume drank in the drink-to-full test. The total amount of food intake as well as the composition of the breakfast did not differ between OBE and NW at admission and discharge and did not change (except a slightly higher fiber intake of OBE at T2). OBE drank more beverages before both appointments resulting in significantly higher total amounts of food and liquids intake. However, none of the breakfast parameters correlated with any EGG data two hours later at baseline, after the stress or the water load test, and therefore did not affect group differences. With respect to the water load test, OBE at T1 drank significantly more water than NW [23], but the ingested water volume did not correlate with any of the EGG data after the water load test, and therefore did not affect group differences.

At admission, OBE reported significantly higher stress ratings after the stress test than NW, and lower stress ratings compared to discharge, which did not differ from NW anymore. Interestingly, higher stress ratings are expected to be related to dysrhythmic GMA such as a decrease of normogastric and an increase of tachgyastric activity due to influences of the ANS in healthy participants [14,29], which was shown by the NW but not by the OBE. However, in our study stress ratings did not correlate with any of the EGG parameters neither in the whole sample nor within groups. As published in a previous article [4] and contradictory to most of the literature in the field [3,7,8,9,10,11], the analysis of parameters of the ANS indicated no differences between OBE at T1 and NW regarding baseline values and reactivity to the stress test: compared to the baseline, both OBE and NW responded to the stress test with a decrease of parasympathetic values (interbeat intervals, IBI; root mean square of successive differences, RMSSD; high frequency normalized units, HFnu) and an increase of sympathetic values (low frequency normalized units, LFnu), which all returned to baseline values at the subsequent recovery period [4]. Because of the sensitivity to speech and movements of EGG recordings, we could analyze only the subsequent recovery period regarding EGG parameters. However, as GMA does not respond as fast as the ANS and as NW’s GMA responses to the stress test could be detected during the recovery period (indicated by the significant difference from OBE), we expect those results can be compared to each other, at least with a time lag. While ANS and GMA seem to be coupled in NW as indicated by a shift towards sympathetic activity during stress and an increase in subsequently recorded tachygastria, these measures seem to be decoupled in OBE as the ANS responds in the same manner as in NW, but not the GMA. Furthermore, GMA seems to be decoupled from the subjective stress response.

These results seem to be contradictory as GMA, ANS, and emotional stress response are considered to be interrelated [14,34]. In the ‘fight or flight’ theory, an increased stress response leads to a shutdown of organ systems which are not directly involved in fight or flight activities which both require additional energy in the peripheral limbs but not in the stomach. To faster supply the limbs with energy and oxygen, heart rate increases and heart rate variability decreases through a shift of the ANS towards an increase in sympathetic activity and a decrease of parasympathetic activity which in turn lead to a disruption of normal GMA with a shift towards dysrhythmic slow waves and tachygastria see [24,25]. Decoupling of GMA and ANS activity has been reported only rarely—e.g., in an experimental study with healthy adults who responded with a reduction of normogastria to both classical music and household noise heard via headphones—but their HRV was not affected by those conditions. Unfortunately, the authors did not provide an explanation for their finding [35].

Finally, most studies reporting altered GMA and GMR in patients with obesity or gastrointestinal disorders have been performed with adults. It may be speculative, but our unexpected finding of a decoupling of GMA and ANS activity could be due to our young sample of children and adolescents in which alterations of those systems have yet not become apparent to the full extent. Long-term studies are needed to investigate the course of alterations in the ANS, GMA, and GMR.

As in every study, some limitations must be mentioned. First, our sex- and age-matched control group was smaller than the group of children with obesity, and the control group was investigated only once. This could impair the comparability of these groups. The control group was tested once only, because no substantial changes were expected in healthy, normal-weight children who did not receive any intervention, and therefore this was considered to be ethically more appropriate. Second, as children had to sit quietly three times for 20 min and EGG recordings are sensitive to various origins of artifacts, there were considerable data dropouts that increased over the course of the investigation. Therefore, data of different children may be included from analysis to analysis. Third, we did not have the opportunity to perform diagnoses of FGIDs according to Rome III or VI criteria, and therefore cannot exclude that some of the OBE were affected by FGIDs. At least, there was no difference in GI symptoms between OBE and NW and symptom scores were low in both groups so that we can assume that our groups were comparable to each other with respect to GI symptoms. Finally, we employed a naturally occurring sample of children with overweight and obesity who had various comorbidities. We cannot rule out that some of these comorbidities, such as psychosomatic disorders, could have an impact on EGG parameters, too, but we had the advantage that we investigated a ‘real world scenario’ with a higher external validity.

## 5. Conclusions

In summary, while GMA of OBE and NW did not differ at baseline, OBE show impaired GMR in response to a stress and a water load task which did not recover during an inpatient weight loss program. Furthermore, GMR seems to be decoupled from a normal subjective stress experience and from ANS reactivity, as shown in a previously published paper [4]. Long-term studies should be performed to further investigate the relationship between GMA, GMR, ANS, and stress experiences in children and adolescents with obesity and the association with the development of gastrointestinal disorders.

## Figures and Tables

**Table 1:** Sample description of children with obesity (OBE) before (T1) and after (T2) weight loss, and of normal-weight control children (NW).

	OBE T1 (*n* = 58)	OBE T2 (*n* = 52)	OBE T1 vs. T2 (*p* Value)	NW (*n* = 26)	OBE T1 vs. NW (*p* Value)
Sex (m:f)	30:28	28:24	n.a.	12:14	0.637
Age (years)	13.05 ± 1.90 (11.8–14.0)	13.10 ± 1.90 (12–14)	n.a.	12.50 ± 0.95 (12–13)	0.134
Height (cm)	163.30 ± 10.48 (156.0–171.5)	163.89 ± 10.07 (156.25–172.5)	n.a.	157.81 ± 9.48 (148.38–165.88)	0.025
Weight (kg)	84.40 ± 20.29 (68.75–99.75)	81.39 ± 19.77 (68.0–97.0)	<0.001	45.29 ± 8.33 (37.15–50.15)	<0.001
BMI (kg/m^2^)	31.29 ± 5.11 (27.38–34.15)	30.06 ± 5.09 (26.0–32.6)	<0.001	18.10 ± 1.63 (16.75–19.05)	<0.001
BMI- z-score	2.52 ± 0.57 (2.1–2.9)	2.36 ± 0.61 (1.9–2.8)	<0.001	−0.17 ± 0.55 (−0.65–0.20)	<0.001
Waist (cm)	104.51 ± 13.28 (94.25–112.50)	101.52 ± 12.80 (93.0–110.0)	<0.001	65.42 ± 5.11 (61.0–70.0)	<0.001
GI symptoms	2.61 ± 2.88 (0.0–4.00)	1.87 ± 2.52 (0.0–3.75)	0.254	2.63 ± 2.53 (0.25–3.75)	0.590

Notes: n.a. = not applicable; All data are presented as mean ± standard deviation and (interquartile range). Data were compared between OBE T1 and OBE T2 (paired *t*-tests or Wilcoxon signed-rank test), and OBE T1 versus NW (Student’s *t*-tests or Mann–Whitney U-test), respectively.

**Table 2:** EGG data of children with obesity before weight loss (OBE T1) and normal-weight control children (NW).

	Mean ± SD		Median (IQR)		FDR-Adjusted
	OBE T1	NW	OBE T1	NW	*p* Value
**Baseline**					
Normogastria	34.1 ± 5.7	33.8 ± 6.9	33.8 (30.1–37.5)	34.4 (29.5–39.3)	0.920
Tachygastria	28.2 ± 5.2	29.7 ± 6.8	29.0 (23.9–32.1)	28.3 (26.3–37.1)	0.636
EGG ratio	1.3 ± 0.5	1.2 ± 0.5	1.2 (1.0–1.4)	1.0 (0.8–1.4)	0.573
**Post stress**					
Normogastria	34.1 ± 6.6	31.6 ± 6.2	33.8 (30.6–38.3)	31.7 (26.7–36.3)	0.311
Tachygastria	27.7 ± 7.2	32.5 ± 5.6	27.3 (24.0–31.3)	33.4 (29.1–35.2)	0.007
EGG ratio	1.3 ± 0.5	1.0 ± 0.3	1.3 (1.0–1.6)	1.0 (0.8–1.1)	0.012
**Post water**					
Normogastria	35.7 ± 6.0	29.1 ± 5.5	36.5 (30.8–39.6)	27.2 (24.8–32.9)	0.004
Tachygastria	27.5 ± 6.8	32.9 ± 3.7	27.1 (23.8–31.8)	32.9 (29.9–36.2)	0.007
EGG ratio	1.5 ± 0.8	0.9 ± 0.2	1.3 (1.0–1.6)	0.9 (0.7–1.0)	0.004

Note: Data are presented as both mean ± standard deviation and median (interquartile range). Group differences were tested with Mann–Whitney U-tests and false discovery rate (FDR)-adjusted *p* values are reported. A FDR < 0.05 was considered statistically significant.

**Table 3:** Hierarchical linear model estimates for the effects of period, group and day on EGG parameters (Model 1: EGG period x group (OBE T1 versus NW), Model 2: period x day in OBE only).

	Normogastria Estimate (SE)	Tachygastria Estimate (SE)	EGG Ratio Estimate (SE)
**Model 1**			
Intercept	33.85 (0.74) ***	29.15 (0.75) ***	1.24 (0.07) ***
Period	−0.77 (0.52)	0.72 (0.53)	−0.04 (0.05)
Group	0.20 (1.47)	1.65 (1.49)	−0.05 (0.13)
Period * Group	−3.24 (1.04) **	2.48 (1.06) *	−0.27 (0.10) *
**Model 2**			
Intercept	33.73 (0.91) ***	28.23 (0.94) ***	1.26 (0.07) ***
Period	0.82 (0.69)	−0.45 (0.70)	0.09 (0.05)
Day	0.16 (1.25)	0.08 (1.27)	0.01 (0.09)
Period * Day	−0.20 (0.99)	1.04 (1.00)	−0.10 (0.07)

Note: Centered predictors. SE, standard error. * *p* < 0.05, ** *p* < 0.01, *** *p* < 0.001.

**Table 4:** EGG data of children with obesity (OBE) before (T1) and after (T2) weight loss.

	Mean ± SD		Median (IQR)		FDR-Adjusted
	OBE T1	OBE T2	OBE T1	OBE T2	*p* Value
**Baseline**					
Normogastria	33.9 ± 6.2	34.4 ± 4.7	33.3 (29.9–37.5)	33.3 (30.3–37.5)	0.857
Tachygastria	27.6 ± 5.3	28.2 ± 5.4	29.0 (23.9–31.6)	29.0 (23.9–31.0)	0.857
EGG ratio	1.3 ± 0.6	1.3 ± 0.4	1.2 (1.0–1.4)	1.2 (1.0–1.4)	0.857
**Post stress**					
Normogastria	33.9 ± 6.7	35.0 ± 6.9	34.1 (30.6–38.3)	34.1 (30.7–38.2)	0.857
Tachygastria	26.8 ± 7.5	28.0 ± 5.4	27.3 (21.3–40.4)	27.3 (22.6–29.5)	0.857
EGG ratio	1.4 ± 0.5	1.3 ± 0.5	1.3 (1.1–1.6)	1.3 (1.1–1.5)	0.857
**Post water**					
Normogastria	35.8 ± 5.9	35.3 ± 11.4	35.5 (31.4–39.5)	35.5 (31.8–39.2)	0.651
Tachygastria	28.0 ± 6.8	30.8 ± 11.4	27.9 (23.3–32.8)	27.9 (23.8–31.7)	0.651
EGG ratio	1.4 ± 0.6	1.2 ± 0.4	1.3 (1.0–1.6)	1.2 (0.9–1.5)	0.651

Notes: Data are presented as both mean ± standard deviation and median (interquartile range). Group differences were tested with Wilcoxon signed-rank tests and false discovery rate (FDR)-adjusted *p* values are reported. A FDR < 0.05 was considered statistically significant.

**Table 5:** Parameters of food intake two hours before the water load test in obese children (OBE) before (T1) and after (T2) weight loss in comparison to normal-weight control children (NW).

	OBE T1	OBE T2	NW	FDR-Adjusted
	Median (IQR)	Median (IQR)	Median (IQR)	*p* Value
Stress rating	3.0 (3.0–4.0)	3.0 (2.0–3.6)	2.4 (1.5–3.1)	OBE T1-NW: 0.005^#^ OBE T2-NW: 0.059^#^ OBE T1-T2: 0.037 ^#^
Amount of food (g)	106 (65–268)	246 (101–326)	243 (89–299)	OBE T1-NW: 0.281 OBE T2-NW: 0.620 OBE T1-T2: 0.036
Amount of liquids (g)	400 (200–500)	400 (200–450)	175 (0–224)	OBE T1-NW: < 0.001 OBE T2-NW: 0.002 OBE T1-T2: 0.036
Total amount of food and liquids (g)	480 (389–679)	547 (313–722)	375 (267–457)	OBE T1-NW: 0.001 OBE T2-NW: 0.002 OBE T1-T2: 0.426
Energy intake (kcal)	281 (202–438)	341 (261–443)	293 (198–490)	OBE T1-NW: 0.754 OBE T2-NW: 0.603 OBE T1-T2: 0.187
Energy density of food (kcal/g)	2.2 (1.3–2.5)	1.5 (1.2–2.6)	1.3 (1.0–1.9)	OBE T1-NW: 0.185 OBE T2-NW: 0.428 OBE T1-T2: 0.885
Fiber intake (g)	4 (2–6)	4 (4–8)	3 (0.3–5)	OBE T1-NW: 0.245 OBE T2-NW: 0.008 OBE T1-T2: 0.036
Carbohydrate intake (g)	40 (28–60)	46 (32–59)	36 (22–66)	OBE T1-NW: 0.847 OBE T2-NW: 0.435 OBE T1-T2: 0.187
Protein intake (g)	10 (5–16)	12 (10–17)	12 (7–15)	OBE T1-NW: 0.718 OBE T2-NW: 0.428 OBE T1-T2: 0.076
Fat intake (g)	9 (4–17)	10 (7–16)	12 (6–17)	OBE T1-NW: 0.675 OBE T2-NW: 0.781 OBE T1-T2: 0.426

Notes: The results are presented as median (interquartile range) due to the non-parametric distribution of the data. Group differences after false discovery rate (FDR) adjustment are presented. A FDR < 0.05 was considered statistically significant. ^#^ *p* values not adjusted due to single comparisons.
